# Management of patients with cervical spine trauma in the emergency department: a systematic critical appraisal of guidelines with a view to developing standardized strategies for clinical practice

**DOI:** 10.1007/s11739-021-02838-1

**Published:** 2021-10-05

**Authors:** Elisa Gesu, Pietro Bellone, Mattia Bonzi, Giulio Andrea Bertani, Barbara Brignolo Ottolini, Paola Bosco, Giorgio Conte, Matteo Ferrari, Elisa Maria Fiorelli, Hayato Kurihara, Monica Solbiati, Luigi Piero Solimeno, Giorgio Costantino

**Affiliations:** 1 Università degli Studi di Milano, Milan, Italy; 2 Emergency Department and Emergency Medicine, Fondazione IRCCS Ca’ Granda Ospedale Maggiore Policlinico, Milan, Italy; 3 Neurosurgery, Fondazione IRCCS Ca’ Granda Ospedale Maggiore Policlinico, Milan, Italy; 4 Neuroradiology, Fondazione IRCCS Ca’ Granda Ospedale Maggiore Policlinico, Milan, Italy; 5 Medical Direction, Fondazione IRCCS Ca’ Granda Ospedale Maggiore Policlinico, Milan, Italy; 6 Internal Medicine Allergology and Immunology, Fondazione IRCCS Ca’ Granda Ospedale Maggiore Policlinico, Milan, Italy; 7 Emergency Surgery and Trauma Unit, IRCCS Humanitas Research Hospital, Rozzano, Milan, Italy; 8 Orthopedics and Traumatology, Fondazione IRCCS Ca’ Granda Ospedale Maggiore Policlinico, Milan, Italy; 9 Health Professions Direction, Fondazione IRCCS Ca’ Granda Ospedale Maggiore Policlinico, Milan, Italy

**Keywords:** Cervical spine, Cervical spine trauma, Emergency Department, Guidelines

## Abstract

Several guidelines on the evaluation of patients with suspected cervical spine trauma in the Emergency Department (ED) exist. High heterogeneity between different guidelines has been reported. Aim of this study was to find areas of agreement and disagreement between guidelines, to identify topics in which further research is needed and to provide an evidence-based cervical spine trauma algorithm for ED physicians. The three most relevant guidelines published on cervical spine trauma in the last 10 years were selected screening websites of the main scientific societies and through the comparison of a normalized Google Scholar and SCOPUS citation index. We compared the selected guidelines through seven a-priori defined questions. In case of disagreement between the guidelines or if the quality of evidence appeared low, evidence from published systematic reviews on the topic was added to build an evidence-based algorithm for approach to spinal trauma in the ED. The three selected guidelines were: NICE 2016, Eastern Association for the Surgery of Trauma 2009 and American Association of Neurological Surgeons and Congress of Neurological Surgeons 2013. We found complete agreement on one question, partial agreement for one questions, no agreement for two questions, while agreement was not assessable for 3 questions. The agreement between different guidelines and the evidence on which recommendations are based is low. An attempt to build an evidence-based algorithm has been made. More studies are needed on many topics.

## Introduction

Trauma is one of the main reasons for patient assessment in emergency departments. Many patients with trauma are suspected of having lesions of the cervical spine [1]. The evaluation and management of patients with cervical spine trauma can be problematic because, on the one hand, missing cervical spine injury (CSI) can have dramatic consequences, and on the other hand, starting an inappropriate diagnostic pathway can be the cause of unnecessary radiation exposure, prolonged emergency department (ED) stay (with the related complications [2] and risk of overcrowding), and over-diagnosis.

Guidelines should help physicians to take decisions that are evidence-based, appropriate and with an explicit evaluation of the risk–benefit ratio. However, guidelines have been deeply criticized. Previous studies have shown that many guidelines are not evidence-based and can be influenced by conflicts of interest. Furthermore, there is often no consensus on the evaluation of the evidence and the suggested recommendations among the various guidelines [3]. The contradictions in guideline recommendations can paradoxically increase the uncertainty of the busy physician.

The aim of this paper was to systematically compare the most important guidelines on cervical spine trauma, to find areas of agreement and disagreement between guidelines, to identify topics in which further research is needed and to provide an evidence-based algorithm with which to approach patients with cervical trauma in the ED.

## Methods

This study is a systematic comparison of secondary evidence.

Briefly, we initially screened the websites of the main medical scientific society interested on the topic, searching for proposed guidelines. Then, a systematic search of the all the available guidelines on cervical spine trauma published in literature was performed, screening the *PubMed* database for key terms referring to guidelines on this topic and choosing the most cited. We lastly asked to experts and looked the references to find further guidelines.

We decided in advance to select up to a maximum of three guidelines for comparison.

Moreover, a search of literature was performed to find any systematic review and metaanlysis on spinal trauma.

The guidelines were selected and compared in terms of seven a-priori emergency department questions. The concordance between different guidelines was evaluated and, in case of discrepancy, the presence of published systematic reviews on the topic was verified in order to find more evidence on the topic. Finally, an algorithm for the management of these patients in the ED was proposed, emphasizing the quality of evidence and the strength of recommendations.

### Guidelines search and selection strategy

We initially searched and included guidelines on the websites of medical scientific societies related to the topic. In particular, we screened the National Institute for Health and Care Excellence (NICE) website, the American College of Physicians (ACP) website, the American College of Emergency Physicians (ACEP) website to include in our study any guideline on cervical spine trauma.

We decided in advance to select up to a maximum of three guidelines for comparison, the number of three has been a-priori chosen to equilibrate the possibility of comparison among them and to permit the readability of the manuscript. Such number has been previously adopted by similar studies [3].

We also decided in advance to perform a guideline search in literature in case of less than three guidelines on the topic were found on medical societies’ websites.

In such case, we then searched the *PubMed* database to find any other guideline on the topic published in the past 10 years. In case of multiple versions of the same guideline, the most recent was considered.

A systematic search of the literature was performed, screening MEDLINE database for the following terms:

(((((spine)

AND

((injury) OR trauma)))

AND

((guideline*) OR guidelines)))

OR

(((spine)

AND

((injury) OR trauma))

AND

Practice Guideline[ptyp]).

Among the results, we included the most cited guidelines in order to reach the number of three guidelines selected for comparison. To compare the citations between guidelines we analysed the guidelines citations on Google Scholar and SCOPUS. We normalized the total number of citations based on the year of publication (number of citations on SCOPUS and Google Scholar divided by the difference between the year of the literature search and year of publication). This method has been previously adopted by similar studies [3].

We lastly asked to experts and looked at references of studies to find any additional relevant guideline.

Two different researchers (EG and PB) independently screened all titles and abstracts. In case of disagreement, a consensus was reached through the consultation of a third researcher (GC).

Last guidelines search was performed in January 2021.

### Literature search

A search aimed to find systematic review and metanalyses on cervical spine trauma was performed, entering in *PubMed* the following keywords “(spine) AND ((injury) OR (trauma))”, filtering results by “*systematic reviews”* and “*metanalysis.*

### A-priori questions

Seven questions, thought to be the most clinically relevant on the topic, were pre-defined as follows: (1) Which patients should be evaluated for traumatic cervical injury? (2) Who should not have cervical spine imaging performed? (3) Who should undergo cervical spine imaging? (4) Which kind of imaging should be performed as an initial investigation? (5) How many and which segments of the cervical spine need to be investigated? (6) Within what timeframe should the imaging report be received? (7) When should the cervical collar be removed?

### Data collection

Two reviewers (PB and EG) independently extracted from the guideline data regarding article title, journal, year of publication, first author, medical society, search strategy, setting of guidelines’ applications, system used to grade the guidelines’ recommendations and the reported references. The answer for each question and the quality of evidence/level of recommendation, according to the method used by the guideline, was extrapolated—if possible—for each stated point.

### Guideline evaluation

Differences and similarities between the selected guidelines were evaluated. Agreement between the guidelines was defined as follows: (1) *no agreement* when the guidelines stated different recommendations; (2) *agreement* when the guidelines suggested the same diagnostic measures or a similar patients’ management strategy; (3) *partial agreement* when different guidelines agreed, with some differences; (4) *not available* when at least one of the guidelines did not address the topic.

In order to facilitate the comparison between the guidelines, the quality of evidence and the strength of recommendation declared from every guideline for each recommendation were extrapolated, if possible, and expressed through the GRADE (Grading of Recommendations, Assessment, Development and Evaluations) system, considering what was expressed by the guidelines’ authors and briefly re-evaluating the references.

If the GRADE system was already used to rank the evidence by the authors, we confirmed their statement.

Briefly, the GRADE system allows grading of the quality of evidence and strength of recommendations. It has the advantages of providing a clear separation between judging confidence in the estimated effect and strength of recommendations and providing a clear, pragmatic interpretation of strong versus weak recommendations. Therefore, unlike many other grading systems, the GRADE approach emphasizes that the strength of a recommendation is affected by factors other than quality of evidence (i.e. values and preferences, costs) [4, 5].

Two different reviewers (PB, EG) independently assessed agreement between guidelines. In case of discrepancy between the two reviewers, a consensus was reached with the contribution of a third reviewer (GC).

In case of agreement between guidelines we provided a pooled rating of recommendations using the GRADE system (see Tables 3 and 4 in the Appendix).

### Literature review and identification of topics for future research

In case of disagreement between the guidelines or if the quality of evidence appeared to be low, we searched for more evidence in systematic reviews on the issue. In case of lacking evidence, we underlined topics needing future research.

### Algorithm development

Starting from the agreement between different guidelines and the evidence from the systematic reviews, using a modified GRADE grading evaluation system, we tried to develop an evidence-based algorithm for the management of cervical spine trauma patients in the ED.

## Results

The medical societies’ websites screening identified only the NICE guideline—entitled “Spinal injury: assessment and initial management” [6]—for inclusion in our study.

**Fig. 1:**
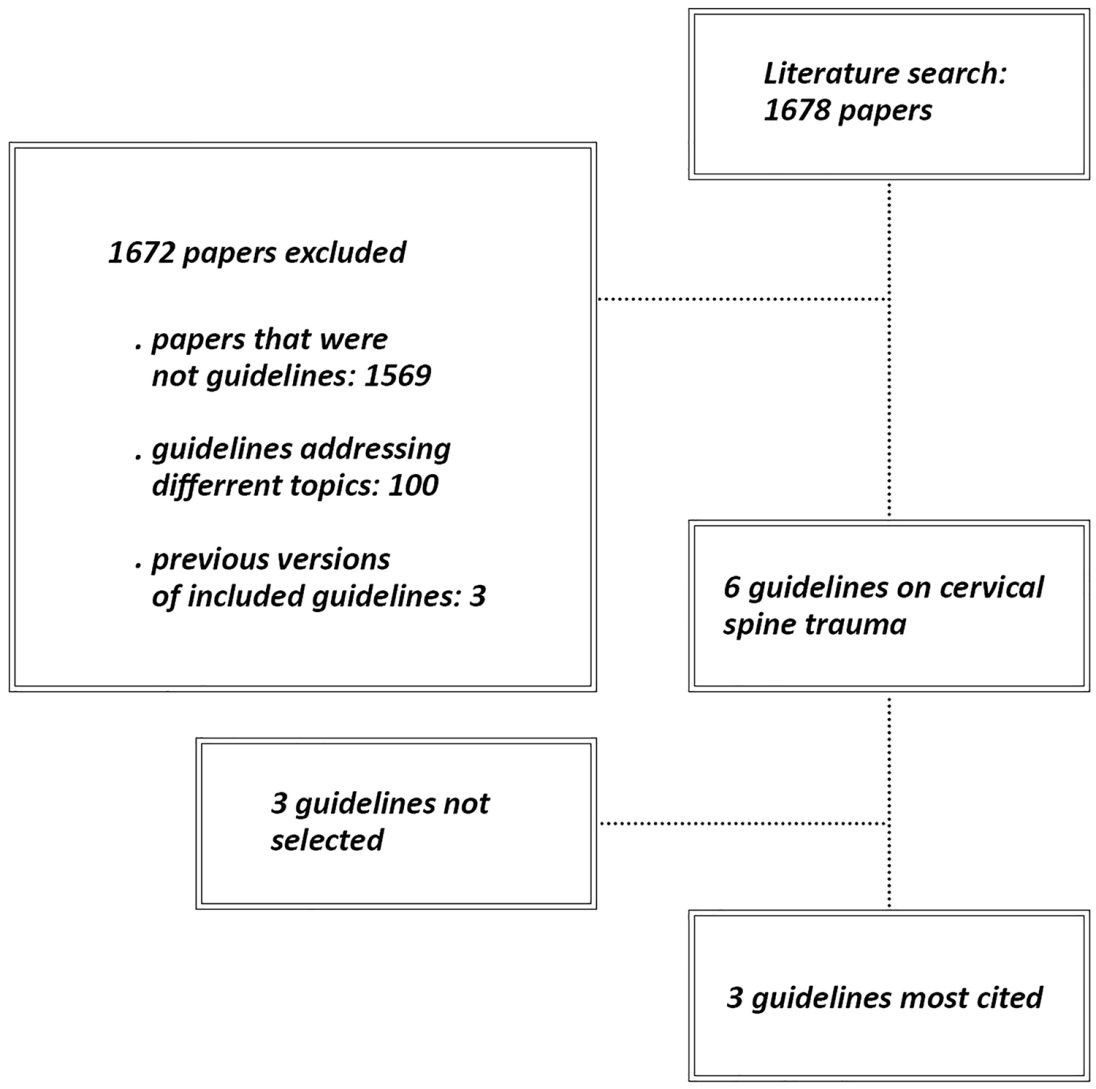
Guidelines selection

The literature search led to 1677 results. After reading titles and abstracts, 1672 were excluded, leaving 5 relevant papers [7, 8, 16–18]. Among these, the two most-cited in the past 10 years [7, 8] were chosen: “Practice Management Guidelines for Identification of Cervical Spine Injuries Following Trauma: Update From the Eastern Association for the Surgery of Trauma Practice Management Guidelines Committee” and “Guidelines for the management of acute cervical spine and spinal cord injuries: 2013 update”. Figure 1 shows the flow chart for study inclusion. Table 1 shows normalized citations for each guideline.

**Table 1:** Normalized citations for each guideline

Guideline	Year of publication	Google Scholar citations	SCOPUS citations	Normalized citations
AANS/CNS	2013	246	199	55.6
EAST	2009	278	202	40
NICE	2016	Website selected	Website selected	Website selected
ACR	2019	14	8	11
Satzherr	2009	34	25	4.92
Georgen	2004	2	2	0.24

The literature screening filtered by systematic reviews and metanalyses led to 1.166 results. Among these, we found relevant in order to add more evidence to our comparison nine studies [1, 2, 9–16].

### Description of the guidelines

Appendix Table 3 shows the main information about the selected guidelines

#### Year of publication and setting

The NICE guidelines were published in 2016. The Eastern Association for the Surgery of Trauma (EAST) Practice Management Guidelines were published in 2009 as an update to the previous EAST guidelines, published in 1998 and 2000. The American Association of Neurological Surgeons and Congress of Neurological Surgeons (AANS/CNS) guidelines were published in 2013 and are an update to the original guideline of 2002. All three guidelines were developed for the ED setting.

#### Grading recommendation system

In the NICE guidelines, the evidence for outcomes from the included studies was evaluated and presented using an adaptation of the GRADE toolbox developed by the international GRADE working group [5].

In the EAST guidelines, articles were classified as Class I, II, or III, as described in the EAST primer on evidence-based medicine: Class I: Prospective, randomized clinical trials; Class II: Clinical studies in which data were collected prospectively or retrospective analyses based on clearly reliable data; Class III: Studies based on retrospectively collected data. The guideline recommendations were then classified as Level 1 (the recommendation is convincingly justifiable based on the available scientific information alone. This recommendation is usually based on Class I data; however, strong Class II evidence may form the basis for a level 1 recommendation); Level 2 (the recommendation is reasonably justifiable by available scientific evidence and strongly supported by expert opinion. This recommendation is usually supported by Class II data or a preponderance of Class III evidence); Level 3 (the recommendation is supported by available data but adequate scientific evidence is lacking. This recommendation is generally supported by Class III data).

In the AANS/CNS, the quality of evidence was assessed as Class I (well-designed and well-executed randomized controlled trials); Class II (comparative studies, including randomized controlled trials with significant flaws, nonrandomized cohort studies, or case–control studies), and Class III (case series and expert opinion). The strength of recommendations is classified as Level I, Level II and Level III.

### Answers and recommendations

Considering all the seven questions, we found some recommendations for all the items. Complete agreement was found only for 1 question. For 3 question agreement was not assessable, while there was no agreement on 2 questions and partial agreement on 1 question. Overall, most of the recommendations were judged as strong through the GRADE system, although mainly founded on low quality of evidence (see Tables 4 and 5 in Appendix).

## Question 1. *Which patients should be evaluated for traumatic cervical injury?*

### Comparison between guidelines

Agreement between the three guidelines was not assessable because only one of the reviewed guidelines (NICE) answers this question, suggesting that all adults who present with suspected spinal column or spinal cord injury secondary to a traumatic event should be evaluated for traumatic cervical injury. Neither the strength of recommendation, the quality of evidence on which this recommendation is based nor the references were clearly reported in the guideline for this indication.

### Literature search and fields for future research

Guidelines do not provide a definition of cervical spine trauma. A traumatic event involves multiple variables, which cannot be standardized under a specific definition. Therefore, the clinician should define whether an event can be considered relevant and harmful to the patient. It is unlikely that future studies could evaluate which kinds of patient should be screened for traumatic cervical spine lesions.

### Considerations

It would be reasonable to state that every trauma patient, with the exception of minor isolated limb lesions, should be clinically evaluated for cervical injuries. It could be assumed that any patient suspected of having cervical spine trauma is eligible for cervical injury screening, from trivial to high-energy traumas. The selection of patients eligible for evaluation for cervical spine trauma remains a decision for the clinician.

## Question 2. *Who should not have cervical spine imaging performed?*

### Comparison between guidelines

All the reviewed guidelines answer this question, but there is no agreement between the three guidelines. Two guidelines (EAST, AANS/CNS) agreed in recommending not to perform imaging in the awake, alert, asymptomatic patient without neurological deficit, without neck pain or tenderness, without distracting injury, who is able to complete a functional range of motion examination. The EAST and the AANS/CNS guidelines refer to the NEXUS (National Emergency X-radiography Utilization Study) algorithm [9] plus evaluation of the cervical range of motion. In contrast, the NICE guidelines require that the Canadian C-spine rule (CCR) be met [10] (Table 2). These two approaches are hardly comparable from a methodological point of view because, while the EAST and the AANS/CNS guidelines use a list of criteria that must all be met, the CCR to which the NICE guidelines refer represents an algorithm with a series of consequential steps which must be progressively respected.

All the guidelines define the strategies on this topic as a strong recommendation. However, the NICE guidelines state that the recommendation is based on very low to low-quality of evidence, EAST guidelines are based on low quality of evidence, while the level of evidence is not assessable in the AANS/CNS guidelines. Of the three guidelines, only the EAST clearly states the references on which the recommendations are based.

### Literature search and fields for future research

Several rules have been proposed for the screening of patients who present in the ED with cervical spine trauma and identification of those who really require imaging investigations. The NEXUS and CCR, the two most important rules on this topic, were published in 2000 and 2001 [9, 10]. The NICE guideline refers to the CCR for the management of patients with cervical spine trauma, while the EAST and the AANS/CNS guidelines use criteria similar to NEXUS. Although there are no extensive validation studies on these rules, the studies upon which they were established enrolled an extremely high number of patients and showed very encouraging results, which has frequently led to their use as a screening tool. However, the two protocols enrolled populations with different characteristics, had different study designs and proposed different approaches. NEXUS was a retrospective study that enrolled all the patients with suspected cervical spine trauma that underwent cervical X-ray by decision of the clinician. The CCR was a prospective study that had several exclusion criteria (in particular, it was not applicable to patients under 18 years) and had strict criteria to identify high-risk patients (in particular, mandatory imaging in patients over 65 years) (Table 2). Both used X-ray as the reference standard to identify cervical spine lesion, while different studies show that computed tomography (CT) has higher diagnostic accuracy and it is now considered the reference standard. In the literature, few validation and comparison studies of the two rules are available [11–14]. The CCR seems to be more accurate than the NEXUS and clinical judgment [15], but further studies are needed.

**Table 2:** NEXUS rule (used by EAST and AANS/CNS guidelines) vs Canadian C-spine rule (used By NICE guideline)

EAST and AANS/CNS guidelines	NICE guidelines (Canadian C-spine rule)
Normal level of alertness	1. High-risk factors that mandates radiography? → If YES perform imaging
Asymptomatic	
Absence of a focal neurologic deficit	Age ≥65 years or
Absence of neck pain tenderness at the posterior midline of the cervical spine	Dangerous mechanism^1^ or
No evidence of intoxication	Paresthesias in extremities
Absence of clinically apparent pain that might distract the patient from the pain of a cervical-spine injury	If NO → following step
Able to complete a functional range of motion Examination	
	2. Low-risk factors that allows safe assessment of range of motion? → If NO perform imaging
	Simple rear-end MVC^2^ or Sitting position in ED or Ambulatory at any time or Delayed onset of neck pain^3^ or Absence of midline C-spine tenderness If YES → following step ↓
	3. Able to actively rotate neck 45° left and right? → if YES → no imaging, remove cervical collar If NO → perform imaging
	^1^Dangerous mechanism: Fall from ≥ 1 m/5 stairs Axial load to head, e.g. diving MVC at high speed (>100 km/h), or with rollover or ejection Motorized recreational vehicle Bicycle collision
	^2^Simple rear-end MVC excludes: Pushed into oncoming traffic Hit by bus/large truck Rollover Hit by high-speed vehicle ^3^Delayed Not immediate onset of neck pain
	Exclusion criteria Patients younger than 16 years; minor injuries, such as simple lacerations, and no suspicion of cervical spine trauma; GCS score < 15; grossly abnormal vital signs; injured more than 48 h previously; penetrating trauma; acute paralysis; known vertebral disease (ankylosing spondylitis, rheumatoid arthritis, spinal stenosis, or previous cervical surgery; patients returned for reassessment of the same injury; pregnancy.

### Considerations

There is not enough evidence to identify a single rule to be applied for patients with cervical spine trauma.

However, we suggest consideration of CCR in addition to clinical judgement to identify which patients with cervical spine trauma should undergo CT scan. Further validation studies on this topic would be useful.

Table 2

## Question 3. *Who should undergo cervical spine imaging?*

### Comparison between guidelines

All the reviewed guidelines answer this question, but there is no agreement between the three guidelines. For this query, between two guidelines (EAST and AANS/CNS) there is substantial agreement recommending imaging in all patients considered difficult to evaluate (obtunded, altered mental status) or symptomatic (the EAST guideline specifies patients with neck pain or tenderness and/or neurological deficit). One guideline (EAST) also recommends imaging in patients with distracting injury. The NICE guideline recommends imaging in all patients with GCS < 15, or at high risk for cervical spine injury according to the CCR or patients at low risk for cervical spine injury according to the CCR but unable to actively rotate their neck 45 degrees left and right. All the guidelines define the strategies on this topic as strong a recommendation. However, the NICE guidelines declare that the recommendation is based on very low to low quality of evidence; the EAST guideline is based on low quality evidence, while the level of evidence is not assessable in the AANS/CNS guideline. None of the three guidelines clearly state the references on which the recommendations are based.

### Literature search and fields for future research

This answer is similar to the previous one. The NICE guidelines set 65 years of age as the cut-off for performing the diagnostic test. Setting an age cut-off could be problematic. While it is reasonable to assume that older age is related to higher risk, further studies should be performed to evaluate the increase in risk of cervical spine injury with age and to assess whether using different age thresholds could be feasible and useful.

### Considerations

Considering that an ideal rule is not available, it could be considered reasonable to perform a CT scan in patients younger than 65 years at high risk, considering CCR and clinical judgment, in polytraumatized patients, in patients older than 65 years that had a cervical spine trauma considered not to be trivial based on clinical judgement (even if asymptomatic).

This suggestion should be considered based on low-quality evidence and future studies are needed to evaluate this topic.

## Question 4. *Which kind of imaging should be performed as an initial investigation?*

### Comparison between guidelines

All the reviewed guidelines answer this question and there is agreement between the three guidelines. All the reviewed guidelines agree recommending CT scan as the first-choice imaging investigation for cervical spine trauma, because of the better sensitivity if compared with X-ray imaging. The AANS/CNS guideline also specifies that if high-quality CT imaging is not available, a 3- view cervical spine series (anteroposterior, lateral, and odontoid views) is recommended. This should be supplemented with CT (when available) if necessary.

All the guidelines define the strategies on this topic as a strong recommendation. However, the NICE guideline states that the recommendation is based on very low to low quality of evidence; the EAST guidelines are based on low quality of evidence, while the level of evidence is not assessable in the AANS/CNS guideline. Of the three guidelines, only the EAST clearly states the references on which the recommendations are based.

### Literature search and fields for future research

Holmes’ meta-analysis showed that CT has better sensitivity than X-ray in patients at high risk (pooled sensitivity of radiography versus CT as 52 percent and 98 percent, respectively [10]). However, it is important to consider which kind of cervical spine lesion could be missed with radiography. Improvement in diagnostic accuracy does not mean improvement in management and outcome for the patient. Even if the segments less evaluable with radiography are those most involved in CSI, it is possible that better sensitivity in diagnosing cervical spine lesions could result in increasing the detection of non-clinically relevant lesions, with no benefit for the patient.

Moreover, the cervical spine rules, on which we have already commented, are based on radiography as the reference standard, and we do not know if replacing radiography with CT could be useful. In conclusion, more studies are needed on the comparison between radiography and spinal CT, setting patient outcomes as the reference standard rather than an abnormality found in the imaging.

### Considerations

Considering the evidence available in the literature, if imaging is considered necessary, CT scan should be performed.

This suggestion should be considered based on low quality evidence.

## Question 5. *How many and which segments of the cervical spine need to be investigated?*

### Comparison between guidelines

Agreement between the three guidelines is not assessable because two of the reviewed guidelines (NICE and AANS/CNS) do not answer this question. The EAST guideline recommends imaging from the occiput to T1. This strategy is defined as a strong recommendation based on low quality of evidence. The references on which the recommendations are based are not clearly stated.

### Literature search and fields for future research

Based on the literature, the most common sites of injury are the second cervical vertebra (C2, or axis, 33%) and the region of the three vertebrae C5, C6, and C7 (50%) [11, 12]. The level of the cervical spine lesion correlates to the severity of the outcome (the higher the lesion, the worse the outcome, from death to quadriplegia, paraplegia or other disabling conditions) [1]. These sites are the most difficult to evaluate with X-ray [15, 16].

### Considerations

If imaging of the cervical spine is necessary, investigation from the occiput to T1 should be performed. This suggestion is based on high-quality evidence and should be considered as a strong recommendation.

## Question 6. *Within what timeframe should the imaging report be received?*

### Comparison between guidelines

Agreement between the three guidelines is not assessable because only the NICE guideline answers this question, recommending that images should be interpreted immediately by a healthcare professional with training and skills in this area. This strategy is defined as a strong recommendation based on very low to low quality of evidence. The references on which the recommendations are based are not clearly stated.

### Literature search and fields for future research

From a clinical point of view, maintaining a cervical collar is associated with negative side effects (risk of decubitus ulcer, worse ventilation) and discomfort for the patient [2]; moreover, a delay in the diagnosis of a severe cervical spine fracture could be associated with worsening of the prognosis. For these reasons, it is desirable that the imaging report be available as soon as possible. No study could address this type of question, and the answer should be based on the opinion of the experts and the hospital organization.

### Considerations

A CT report should be obtained as soon as possible (ideally within one hour). This suggestion is based on low-quality evidence and should be considered as a weak recommendation.

## Question 7. *When should the cervical collar be removed?*

### Comparison between guidelines

All the reviewed guidelines answer this question and there is partial agreement between the three guidelines. Two guidelines (EAST and AANS/CNS) agree in recommending to remove the cervical collar in the awake, alert, asymptomatic patient without neurological deficit, without neck pain or tenderness, without distracting injury, who is able to complete a functional range of motion examination. These two guidelines agree even in recommending to consider continuing cervical immobilization until the patient is asymptomatic or to remove the cervical collar after a negative MRI or after adequate flexion–extension X-ray, in the awake, symptomatic patient with a negative CT scan. Moreover, the AANS/CNS guideline suggests possibly discontinuing cervical immobilization at the discretion of the treating physician. Only one guideline (EAST) considers the obtunded patient with a negative CT scan and gross motor function of all four extremities, recommending to continue cervical collar immobilization until a clinical examination can be performed or to remove the cervical collar on the basis of CT alone or plus MRI and, if MRI shows nothing abnormal, to remove the cervical collar. The NICE guidelines require patients to have low- risk factors for cervical spine injury as identified and indicated by the CCR, to be pain free and able

to actively rotate the neck 45 degrees left and right. The strength of the recommendations and quality of evidence on which these are based are shown in Table 1.

### Literature search and fields for future research

It is safe to remove the cervical collar if the patient is asymptomatic and the imaging examination is negative. The literature is not clear about which management strategy could be the best in patients who are still symptomatic but have negative imaging.

### Considerations

In clinical practice, the cervical collar can probably be safely removed in asymptomatic patients and in patients who are clinically evaluable, with have no neurological deficits and with negative CT imaging. In special situations the clinician should evaluate case-by-case if maintaining cervical collar and/or perform more investigations. In doubtful situations, spine specialist consultation may be considered. This suggestion is based on low-quality evidence and should be considered as a weak recommendation*.*

## Discussion

The aim of this paper was to compare the main guidelines in the literature on cervical spine trauma evaluation and management, to identify topics in which further research is needed, to extrapolate recommendations on several topics of interest and to provide, if possible, a univocal pathway that can help clinicians in approaching patients with suspected cervical trauma/injury. Evaluating the answers to our a-priori questions in the selected guidelines, it emerged that there was very scarce agreement between different guidelines. In particular, for only one question (“which kind of imaging should be performed as an initial investigation?”, question 4) there was complete agreement in the answer. For one other question there was only partial agreement, for two questions there was no agreement, while for three questions it was not possible to establish any agreement because some guidelines did not cover the topic. This underlines that between the different guidelines there is wide inhomogeneity, not only between what is recommended but also in the covered topics. This fact could perhaps be explained by the fact that even when the same question is answered, the cited references often differ or, if the references are similar, the grade of recommendation can differ. Moreover, most of the recommendations are judged to be of low quality.

The guidelines use different criteria to evaluate the quality of evidence (QoL) and to state the strength of recommendation. In particular, the NICE guidelines state that they used the GRADE method, while the other two use their own tailored criteria. The GRADE, among those currently available, is a codified and reproducible evaluation system for the quality of the evidence and strength of the recommendations, but was published in 2013, after the AANS/CNS and the EAST guidelines.

The inhomogeneity between the grading system for QoL and strength of recommendations could represent an obstacle for the clinician examining several different guidelines and needing to identify the most useful one.

The analysis of the guidelines and the subsequent search of the available literature highlight that further studies are needed for multiple fields of research. In particular, more studies would be useful for the following: (1) comparing the available tools to screen high-risk patients for cervical spine injury with the purpose of identifying the most accurate one and definitively validating it, setting CT scan as the reference standard; (2) evaluating the increase in risk of cervical spine injury with age and the utility of different age thresholds; (3) comparing the diagnostic accuracy of spinal CT and X-ray, setting some fixed outcome (based on the impact on the patient, i.e. need for surgery) as the reference standard; (4) evaluating the best management for patients who are still symptomatic after negative imaging.

We ultimately proposed an evidence-based algorithm for the management of patients with cervical spine trauma.

This study has several limitations. Our comparison approached only three guidelines—the most cited—which were published in different years with a time interval of 9 years between the oldest and the newest.

We selected the three most-cited guidelines, but these criteria do not necessarily guarantee that the suggested recommendations are the best available.

Our protocol is an attempt to help the clinician based on these guidelines, the available evidence-based information and our clinical experience and should be validated in the future.

## Conclusions

**Fig. 2:**
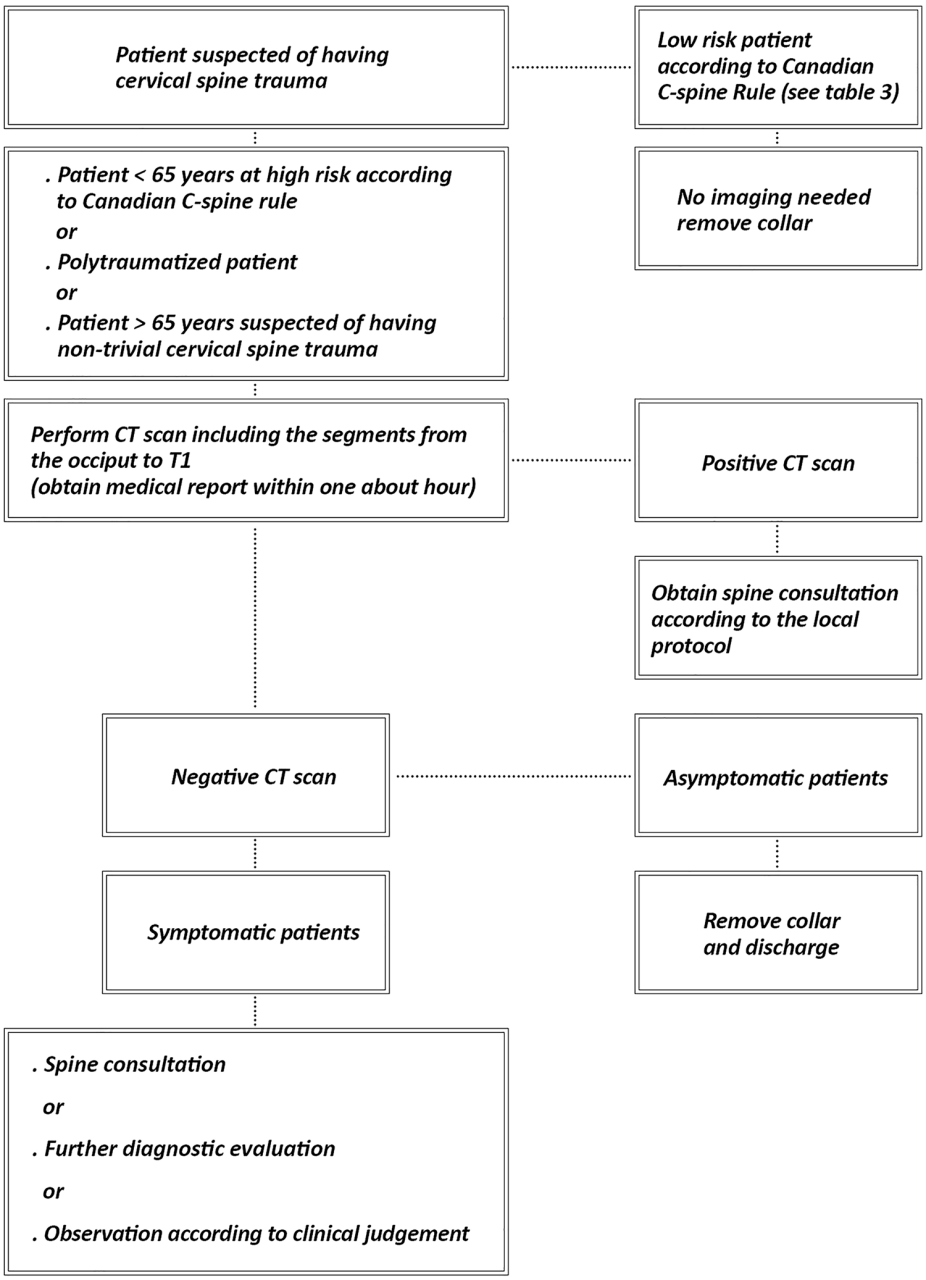
Proposed algorithm for management of patients suspected for cervical spine trauma

There is scarce agreement between the guidelines addressing cervical spine trauma in the ED. Further research is needed to evaluate the best management of these patients and to identify patients that need to undergo specific diagnostic evaluation. Based on our comparison of the guidelines and literature search, we have proposed an evidence-based algorithm for the management of cervical spine trauma patients in the ED (Fig. 2).

## Appendix

See Tables 3, 4 and 5.

**Table 3:** Normalized citations for each guideline

Guidelines	Website/Journal	Grading recommendation system	Search strategy
EAST 2009	The Journal of TRAUMA^®^ Injury, Infection, and Critical Care	Articles were classified as Class I, II, or III as described in the EAST primer on evidence-based medicine as follows: Class I: Prospective, randomized clinical trials (no references) Class II: Clinical studies in which data were collected prospectively or retrospective analyses based on clearly reliable data (20 references) Class III: Studies based on retrospectively collected data (32 references). Recommendations were classified as levels 1, 2, or 3 according to the following definitions: Level 1: The recommendation is convincingly justifiable based on the available scientific information alone. This recommendation is usually based on class I data; however, strong class II evidence may form the basis for a level 1 recommendation, especially if the issue does not lend itself to testing in a randomized format. Conversely, low quality or contradictory class I data may not be able to support a level 1 recommendation Level 2: The recommendation is reasonably justifiable by available scientific evidence and strongly supported by expert opinion. This recommendation is usually supported by class II data or a preponderance of class III evidence Level 3: The recommendation is supported by available data, but adequate scientific evidence is lacking. This recommendation is generally supported by class III data. This type of recommendation is useful for educational purposes and in guiding future clinical research	A search of the National Library of Medicine and the National Institutes of Health MEDLINE database was performed using PubMed (http://www.pubmed.gov). The search retrieved English language articles regarding the identification of CS injury from 1998 to 2007; review articles, letters to the editor, editorials, other items of general commentary, and case reports were excluded from the search. These articles were then reviewed for relevance by the committee chair, and the final reference list of 78 citations was distributed to the remainder of the study group for review. Of these, 52 were felt to be useful for construction of these guidelines, and an evidentiary table was constructed
AANS/CNS 2013	Clinical Neurosurgery	Different from previous recommendations, the levels that used to be called standards, guidelines, and options are now referred to as Level I, Level II, and Level III, bringing them more in line with other neurosurgical and medical specialty paradigms and allowing the use of the term guidelines to denote the broader scope of the overall recommendations	In accordance with the established practice of guideline development within organized neurosurgery, a thorough review of the medical literature was undertaken for each subject chosen for evaluation. Although literature outside the English language was excluded, a sample of non-English abstracts that could be found in the database of the National Library of Medicine failed to reveal any data significantly different from what we found in the English literature. Each chapter of recommendations contained in the new guidelines uses standard search techniques fully described in each chapter After articles appropriate to each review question were identified, a rigorous critical evaluation was undertaken to establish the strength (quality) of the evidence and the level (certainty) of the recommendations
NICE 2016	https://www.nice.org.uk/guidance/ng41	GRADE	Searches were undertaken according to the parameters stipulated within the NICE Guidelines Manual [2012]. Databases were searched using medical subject headings and free-text terms. Foreign language studies were not reviewed and, where possible, searches were restricted to articles published in the English language. All searches were conducted in MEDLINE, Embase, and the Cochrane Library, and were updated for the final time on 27th March 2015. No papers added to the databases after this date were considered Search strategies were quality assured by cross-checking reference lists of highly relevant papers, analysing search strategies in other systematic reviews, and asking GDG members to highlight any additional studies. The questions, the study types applied, the databases searched and the years covered can be found in Appendix F. The titles and abstracts of records retrieved by the searches were sifted for relevance, with potentially significant publications obtained in full text. These were then assessed against the inclusion criteria

COR: class of recommendation

LOE: level of evidence

**Table 4:** Agreement between guidelines

Question 1. *Which patients should be evaluated for traumatic cervical injury?*			
NICE 2016	EAST 2009	AANS/CNS 2013	Agreement between guidelines and pooled recommendation
All adults who present with suspected spinal column or spinal cord injury secondary to a traumatic event	Not covered by guidelines	Not covered by guidelines	Not assessable (NA)
Original GRADE: NA	Original COR/LOE: NA	Original COR/LOE: NA	
Extrapolated GRADE: NA	Extrapolated GRADE: NA	Extrapolated GRADE: NA	GRADE of the pooled recommendation: NA

*Question 2. Who should not have cervical spine imaging performed?*			
NICE 2016	EAST 2009	AANS/CNS 2013	Agreement between guidelines and pooled recommendation
Do not carry out or maintain full in-line spinal immobilisation or request imaging for people if: they have low-risk factors for cervical spine injury as identified and indicated by the Canadian C-spine rule, are pain free and are able to actively rotate their neck 45 degrees left and right. Assess whether the person is at high, low or no risk for cervical spine injury using the Canadian C-spine rule as follows: the person is at high risk if they have at least one of the following high-risk factors: age 65 years or older dangerous mechanism of injury (fall from a height of greater than 1 m or 5 steps, axial load to the head—for example diving, high-speed motor vehicle collision, rollover motor accident, ejection from a motor vehicle, accident involving motorised recreational vehicles, bicycle collision, horse riding accidents) paraesthesia in the upper or lower limbs the person is at low risk if they have at least one of the following low-risk factors: involved in a minor rear-end motor vehicle collision comfortable in a sitting position ambulatory at any time since the injury no midline cervical spine tenderness delayed onset of neck pain the person remains at low risk if they are the following: unable to actively rotate their neck 45 degrees to the left and right (the range of the neck can only be assessed safely if the person is at low risk and there are no high-risk factors) the person has no risk if they—have one of the above low-risk factors and—are able to actively rotate their neck 45 degrees to the left and right	In awake, alert patients with trauma without neurologic deficit or distracting injury who have no neck pain or tenderness with full range of motion of the CS: CS imaging is not necessary and the cervical collar may be removed	In the awake, asymptomatic patient who is without neck pain or tenderness, who has a normal neurological examination, who is without an injury detracting from an accurate evaluation, and who is able to complete a functional range of motion examination, radiographic evaluation of the cervical spine is not recommended	NO AGREEMENT
Original GRADE: strong recommendation, very low to low quality of evidence	Original COR/LOE: level 2	Original COR/LOE: level 1	
Extrapolated GRADE: strong recommendation, very low to low quality of evidence	Extrapolated GRADE: strong recommendation, low quality of evidence	Extrapolated GRADE: strong recommendation, quality of evidence NA	GRADE of the pooled recommendation: NA

*Question 3. Who should undergo cervical spine imaging?*			
NICE 2016	EAST 2009	AANS/CNS 2013	Agreement between guidelines and pooled recommendation
Carry out or maintain full in-line spinal immobilisation and request imaging if: a high-risk factor for cervical spine injury is identified and indicated by the Canadian C-spine rule or a low-risk factor for cervical spine injury is identified and indicated by the Canadian C-spine rule and the person is unable to actively rotate their neck 45 degrees left and right	All patients in whom CS injury is suspected must have radiographic evaluation. This applies to patients with pain or tenderness, patients with neurologic deficit, patients with altered mental status, and patients with distracting injury	In the awake, symptomatic patient, high-quality CT imaging of the cervical spine is recommended. In the obtunded or unevaluable patient, high-quality CT imaging is recommended as the initial imaging modality of choice	NO AGREEMENT
Original GRADE: strong recommendation, very low to low quality of evidence	Original COR/LOE: level 2	Original COR/LOE: level 1	
Extrapolated GRADE: strong recommendation, very low to low quality of evidence	Extrapolated GRADE: strong recommendation, low quality of evidence	Extrapolated GRADE: strong recommendation, quality of evidence NA	GRADE of the pooled recommendation: NA

*Question 4. Which kind of imaging should be performed as an initial investigation?*			
NICE 2016	EAST 2009	AANS/CNS 2013	Agreement between guidelines and pooled recommendation
Perform CT in adults (16 or over) if imaging for cervical spine injury is indicated by the Canadian C-Spine rule	The primary screening modality is axial CT from the occiput to T1 with sagittal and coronal reconstructions Plain radiographs contribute no additional information and should not be obtained	In the awake, symptomatic patient, high-quality CT imaging of the cervical spine is recommended. If high-quality CT imaging is available, routine 3-view cervical spine radiographs are not recommended If high-quality CT imaging is not available, a 3-view cervical spine series (anteroposterior, lateral, and odontoid views) is recommended. This should be supplemented with CT (when it becomes available) if necessary to further define areas that are suspicious or not well visualized on the plain cervical X-rays. In the obtunded or unevaluable patient, high-quality CT imaging is recommended as the initial imaging modality of choice. If CT imaging is available, routine 3-view cervical spine radiographs are not recommended. If high-quality CT imaging is not available, a 3-view cervical spine series (anteroposterior, lateral, and odontoid views) is recommended. This should be supplemented with CT (when it becomes available) if necessary to further define areas that are suspicious or not well visualized on the plain cervical X-rays	AGREEMENT CT scan is recommended as initial imaging investigation
Original GRADE: strong recommendation, very low to low quality of evidence	Original COR/LOE: level 2	Original COR/LOE: level 1	
Extrapolated GRADE: strong recommendation, very low to low quality of evidence	Extrapolated GRADE: strong recommendation, low quality of evidence	Extrapolated GRADE: strong recommendation, quality of evidence NA	GRADE of the pooled recommendation: strong recommendation, low quality of evidence

*Question 5. How many and which segments of the cervical spine need to be investigated?*			
NICE 2016	EAST 2009	AANS/CNS 2013	Agreement between guidelines and pooled recommendation
Not covered by guidelines	CT CS must include axial images from the occiput to T1 with sagittal and coronal reconstructions	Not covered by guidelines	NA
Original GRADE: NA	Original COR/LOE: level 2	Original COR/LOE: NA	
Extrapolated GRADE: NA	Extrapolated GRADE: strong recommendation, low quality of evidence	Extrapolated GRADE: NA	GRADE of the pooled recommendation: NA

**Table:**

*Question 6.* *Within what timeframe should the imaging report be received?*			
NICE 2016	EAST 2009	AANS/CNS 2013	Agreement between guidelines and pooled recommendation
Imaging for spinal injury should be performed urgently, and the images should be interpreted immediately by a healthcare professional with training and skills in this area	Not covered by guidelines	Not covered by guidelines	NA
Original GRADE: weak recommendation, very low to low quality of evidence	Original COR/LOE: NA	Original COR/LOE: NA	
Extrapolated GRADE: weak recommendation, very low to low quality of evidence	Extrapolated GRADE: NA	Extrapolated GRADE: NA	GRADE of the pooled recommendation: NA

*Question 7.* *When should the cervical collar be removed?*			
NICE 2016	EAST 2009	AANS/CNS 2013	Agreement between guidelines and pooled recommendation
Do not carry out or maintain full in-line spinal immobilisation or request imaging for people if: they have low-risk factors for cervical spine injury as identified and indicated by the Canadian C-spine rule, are pain free and are able to actively rotate their neck 45 degrees left and right	Cervical collar should be removed as soon as feasible after trauma. (level 3) In awake, alert patients with trauma without neurologic deficit or distracting injury who have no neck pain or tenderness with full range of motion of the CS imaging is not necessary and the cervical collar may be removed. (level2) For the neurologically intact awake and alert patient complaining of neck pain with a negative CT: A. Continue cervical collar B. Cervical collar may be removed after negative MRI (level 3) C. Cervical collar may be removed after negative and adequate F/E films (level 3) For the obtunded patient with a negative CT and gross motor function of all four extremities the risk/benefit ratio of obtaining MRI in addition to CT is not clear, and its use must be individualized in each institution (level 3). Options are as follows: A. Continue cervical collar immobilization until a clinical examination can be performed B. Remove the cervical collar on the basis of CT alone C. Obtain MRI If MRI disclosed nothing abnormal, the cervical collar may be safely removed (level 2)	In the awake, asymptomatic patient who is without neck pain or tenderness, who has a normal neurological examination, who is without an injury detracting from an accurate evaluation, and who is able to complete a functional range of motion examination, radiographic evaluation of the cervical spine is not recommended. Discontinuance of cervical immobilization for these patients is recommended without cervical spinal imaging In the awake patient with neck pain or tenderness and normal high-quality CT imaging or normal 3-view cervical spine series (with supplemental CT if indicated), the following recommendations should be considered: (1) Continue cervical immobilization until asymptomatic; (2) Discontinue cervical immobilization after normal and adequate dynamic flexion/extension radiographs; (3) Discontinue cervical immobilization after a normal MRI obtained within 48 h of injury (4) Discontinue cervical immobilization at the discretion of the treating physician	PARTIAL AGREEMENT Cervical collar should be removed in awake, alert, asymptomatic patient without neurologic deficit, without neck pain or tenderness, without distracting injury, who is able to complete a functional range of motion examination (EAST; AANS/CNS) and who have low-risk factors for cervical spine injury as identified by the Canadian C-spine rule (NICE—see NICE recommendation) In the awake symptomatic patient with a negative CT scan consider: Continue cervical collar until asymptomatic (EAST, AANS/CNS) Remove cervical collar after a negative MRI (EAST, AANS/CNS) Remove cervical collar after adequate flexion–extension X-ray (EAST, AANS/CNS) Discontinue cervical immobilization at the discretion of the treating physician (AANS/CNS) For the obtunded patient with a negative CT and gross motor function of all four extremities: Continue cervical collar immobilization until a clinical examination can be performed Remove the cervical collar on the basis of CT alone Obtain MRI If MRI disclosed nothing abnormal, the cervical collar may be safely removed (EAST)
Original GRADE: strong recommendation, very low to low quality of evidence	Original COR/LOE: (see in the text above) level 3, level 2, level 3, level 3, level 3, level 2	Original COR/LOE: level 1; level 3	
Extrapolated GRADE: strong recommendation, very low to low quality of evidence	Extrapolated GRADE: Level 2 here evaluated as follows: “strong recommendation, low quality of evidence” Level 3 here evaluated as “weak recommendation, low quality of evidence”	Extrapolated GRADE: strong recommendation, quality of evidence NA; weak recommendation, quality of evidence NA	GRADE of the pooled recommendation: NA

**Table 5:** References for each guideline

Question 1: *Which kind of patients should be evaluated for traumatic cervical injury?*		
NICE 2016	EAST 2009	AANS/CNS 2013
Page: 17 References: NA	Page: NA References: NA	Page: NA References: NA

Question 2. *Who should not have cervical spine imaging performed?*		
NICE 2016	EAST 2009	AANS/CNS 2013
Page: 72, 73 References: NA	Page: 654 References Bachulis BL, Long WB, Hynes JD, Johnson MC. Clinical indications for cervical spine radiographs in the traumatized patient. Am J Surg. 1987; 153:473–478 Ersoy G, Karcioglu O, Enginbas Y, Eray O, Ayrik C. Are cervical spine X-rays mandatory in all blunt trauma patients? Eur J Emerg Med. 1995;2:191–195 Fischer RP. Cervical radiographic evaluation of alert patients following blunt trauma. Ann Emerg Med. 1984;13:905–907 Hoffman JR, Schriger DL, Mower WR, Luo JS, Zucker M. Low-risk criteria for cervical spine radiography in blunt trauma: a prospective study. Ann Emerg Med. 1992;12:1454–1460 Kreipke DL, Gillespie KR, McCarthy MC, Mail JT, Lappas JC, Broadie TA. Reliability of indications for cervical spine films in trauma patients. J Trauma. 1989;29:1438–1439 Lindsey RW, Diliberti TC, Doherty BJ, Watson AB. Efficacy of radiographic evaluation of the cervical spine in emergency situations. South Med J. 1993;86:1253–1255 Neifeld GL, Keene JG, Hevesy G, Leikin J, Proust A, Thisted RA. Cervical injury in head trauma. J Emerg Med. 1988;6:203–207 Roberge RJ, Wears RC, Kelly M, et al. Selective application of cervical spine radiography in alert victims of blunt trauma: a prospective study. J Trauma. 1988;28:784–788 Roth BJ, Martin RR, Foley K, Barcia PJ, Kennedy P. Roentgenographic evaluation of the cervical spine. A selective approach. Arch Surg. 1994;129:643–645 Saddison D, Vanek VW, Racanelli JL. Clinical indications for Cervical spine radiographs in alert trauma patients. Am Surg. 1991; 57:366–369 Velmahos GC, Theodorou D, Tatevossian R, et al. Radiographic cervical spine evaluation in the alert asymptomatic blunt trauma victim: much ado about nothing. J Trauma. 1996;40:768–774 Gonzales RP, Fried PO, Bukhalo M, Holevar MR, Falimirski ME. Roleof clinical examination in screening for blunt cervical spine injury. J Am Coll Surg. 1999;189:152–157 Hoffman JR, Mower WR, Wolfson AB, Todd KH, Zucker MI. Validation of a set of clinical criteria to rule out injury to the cervical spine in patients with blunt trauma. N Engl J Med. 2000;343:94–99 Stiell IG, Clement CM, McKnight RD, et al. The Canadian C-spine rule versus the NEXUS low-risk criteria in patients with trauma. N Engl J Med. 2003;349:2510–2518 Duane TM, Dechert T, Wolfe LG, Aboutanos MB, Malhotra AK, Ivatury RR. Clinical examination and its reliability in identifying cervical spine fractures. J Trauma. 2007;62:1405–1410	Page: 83 References: NA

Question 3. *Who should undergo cervical spine imaging?*		
NICE 2016	EAST 2009	AANS/CNS 2013
Page: 73, 74 References: NA	Page: 654 References: NA	Page: 84, 85 References: NA

Question 4. *Which kind of imaging should be performed as an initial investigation?*		
NICE 2016	EAST 2009	AANS/CNS 2013
Page: 148 References: NA	Page: 654 References Berne JD, Velmahos GC, El-Tawil Q, et al. Value of complete cervical helical computed tomographic scanning in identifying cervical spine injury in the unevaluable blunt trauma patient with multiple injuries: a prospective study. J Trauma. 1999;47:896–903 Griffen MM, Frykberg ER, Kerwin AJ, et al. Radiographic clearance of blunt cervical spine injury: plain radiograph or computed tomography scan? J Trauma. 2003;55:222–227 Diaz JJ, Gillman C, Morris JA Jr, May AK, Carrillo YM, Guy J. Are five-view plain films of the cervical spine unreliable? A prospective evaluation in blunt trauma patients with altered mental status. J Trauma 2003;55:658–664 Brohi K, Healy M, Fotheringham T, et al. Helical computed tomographic scanning for the evaluation of the cervical spine in the unconscious, intubated trauma patient. J Trauma. 2005;58:897–901 Mathen R, Inaba K, Munera F, et al. Prospective evaluation of multislice computed tomography versus plain radiographic cervical spine clearance in trauma patients. J Trauma. 2007;62:1427–1431 Holmes JF, Akkinepalli R. Computed tomography versus plain radiography to screen for cervical spine injury: a meta-analysis. J Trauma. 2005;58:902–905 Barba CA, Taggert J, Morgan AS, et al. A new cervical spine clearance protocol using computed tomography. J Trauma. 2001;51:652–657 Brown CV, Antevil JL, Sise MJ, Sack DI. Spiral computed tomography for the diagnosis of cervical, thoracic, and lumbar spine fractures: its time has come. J Trauma. 2005;58:890–896 Rabb CH, Johnson JL, VanSickle D, Beauchamp K, Bolles G, Moore EE. Are upright lateral cervical radiographs in the obtunded trauma patient useful? A retrospective study. World J Emerg Surg. 2007;2:4 Sanchez B, Waxman K, Jones T, Conner S, Chung R, Becerra S Cervical spine clearance in blunt trauma: evaluation of a computed tomography-based protocol. J Trauma. 2005;59:179–183 Schenarts PJ, Diaz J, Kaiser C, Carrillo Y, Eddy V, Morris JA Jr Prospective comparison of admission computed tomographic scan and plain films of the upper cervical spine in trauma patients with altered mental status. J Trauma. 2001;51:663–669 Widder S, Doig C, Burrowes P, Larsen G, Hurlbert RJ, Kortbeek JB. Prospective evaluation of computed tomographic scanning for the spinal clearance of obtunded trauma patients: preliminary results. J Trauma. 2004;56:1179–1184 Daffner RH. Cervical radiography for trauma patients: a time effective technique? AJR Am J Roentgenol. 2000;175:1309–1311 Daffner RH. Helical CT of the cervical spine for trauma patients: a time study. AJR Am J Roentgenol. 2001;177:677–679 Blackmore CC, Ramsey SD, Mann FA, Deyo RA. Cervical spine screening with CT in trauma patients: a cost-effectiveness analysis. Radiology. 1999;212:117–125	Page: 84, 85 References: NA

Question 5. *How many and which segments of the cervical spine need to be investigated?*		
NICE 2016	EAST 2009	AANS/CNS 2013
Page: NA References: NA	Page:654 References: NA	Page: NA References: NA

Question 6. *Within what timeframe should the imaging report be received?*		
NICE 2016	EAST 2009	AANS/CNS 2013
Page: 147 References: NA	Page: NA References: NA	Page: NA References: NA

Question 7. *When should the cervical collar be removed?*		
NICE 2016	EAST 2009	AANS/CNS 2013
Page: 73 References: NA	Page: 654 References Ackland HM, Cooper JD, Malham GM, Kossmann T. Factors predicting cervical collar-related decubitus ulceration in major trauma patients. Spine. 2007;32:423–428 Chendrasekhar A, Moorman DW, Timberlake GA. An evaluation of the effects of semirigid cervical collars in patients with severe closed head injury. Am Surg. 1998;64:604–606 Powers J, Daniels D, McGuire C, Hilbish C. The incidence of skin breakdown associated with the use of cervical collars. J Trauma Nurs. 2006;13:198–200 Hunt K, Hallworth S, Smith M. The effects of rigid collar placement on intracranial and cerebral perfusion pressures. Anaesthesia. 2001;56:511–513 Mobbs RJ, Stoodley MA, Fuller J. Effect of cervical hard collar on intracranial injury after head injury. ANZ J Surg. 2002;72:389–391 Stelfox HT, Velmahos GC, Gettings E, Bigatello LM, Schmidt U. Computed tomography for early and safe discontinuation of cervical spine immobilization in obtunded multiply injured patients. J Trauma. 2007;63:630–636.14. Bachulis BL, Long WB, Hynes JD, Johnson MC. Clinical indications for cervical spine radiographs in the traumatized patient. Am J Surg. 1987;153:473–478 Ersoy G, Karcioglu O, Enginbas Y, Eray O, Ayrik C. Are cervical spine X-rays mandatory in all blunt trauma patients? Eur J Emerg Med. 1995;2:191–195 Fischer RP. Cervical radiographic evaluation of alert patients following blunt trauma. Ann Emerg Med. 1984;13:905–907 Hoffman JR, Schriger DL, Mower WR, Luo JS, Zucker M. Low-risk criteria for cervical spine radiography in blunt trauma: a prospective study. Ann Emerg Med. 1992;12:1454–1460 Kreipke DL, Gillespie KR, McCarthy MC, Mail JT, Lappas JC, Broadie TA. Reliability of indications for cervical spine films in trauma patients. J Trauma. 1989;29:1438–1439 Lindsey RW, Diliberti TC, Doherty BJ, Watson AB. Efficacy of radiographic evaluation of the cervical spine in emergency situations. South Med J. 1993;86:1253–1255 Neifeld GL, Keene JG, Hevesy G, Leikin J, Proust A, Thisted RA Cervical injury in head trauma. J Emerg Med. 1988;6:203–207 Roberge RJ, Wears RC, Kelly M, et al. Selective application of cervical spine radiography in alert victims of blunt trauma: a prospective study. J Trauma. 1988;28:784–788 Roth BJ, Martin RR, Foley K, Barcia PJ, Kennedy P. Roentgenographic evaluation of the cervical spine. A selective approach. Arch Surg. 1994;129:643–645 Saddison D, Vanek VW, Racanelli JL. Clinical indications for cervical spine radiographs in alert trauma patients. Am Surg. 1991; 57:366–369 Velmahos GC, Theodorou D, Tatevossian R, et al. Radiographic cervical spine evaluation in the alert asymptomatic blunt trauma victim: much ado about nothing. J Trauma. 1996;40:768–774 Gonzales RP, Fried PO, Bukhalo M, Holevar MR, Falimirski ME. Role of clinical examination in screening for blunt cervical spine injury. J Am Coll Surg. 1999;189:152–157 Hoffman JR, Mower WR, Wolfson AB, Todd KH, Zucker MI. Validation of a set of clinical criteria to rule out injury to the cervical spine in patients with blunt trauma. N Engl J Med. 2000;343:94–99 Stiell IG, Clement CM, McKnight RD, et al. The Canadian C-spine rule versus the NEXUS low-risk criteria in patients with trauma. N Engl J Med. 2003;349:2510–2518 Schuster R, Waxman K, Sanchez B, et al. Magnetic resonance imaging is not needed to clear cervical spines in blunt trauma patients with normal computed tomographic results and no motor deficits. Arch Surg. 2005;140:762–766 Fazl M, LaFebvre J, Willinsky RA, Gertzbein S. Posttraumatic ligamentous disruption of the cervical spine, an easily overlooked diagnosis: presentation of three cases. Neurosurgery. 1990;26:764–678 Ficker R, Gachter A. Lateral flexion/extension radiographs: still recommended following cervical spine injury. Arch Orthop Trauma Surg 1994;113:115–116 Lewis LM, Docherty M, Ruoff BE, Fortney JP, Keltner RA Jr, Britton P. Flexion–extension views in the evaluation of cervical spine injuries. Ann Emerg Med. 1991;20:117–121 Brady WJ, Moghtader J, Cutcher D, Exline C, Young J. ED use of flexion–extension cervical spine radiography in the evaluation of blunt trauma. Am J Emerg Med. 1999;17:504–508 Insko EK, Gracias VH, Gupta R, Goettler CE, Gaieski DF, Dalinka MK. Utility of flexion and extension radiographs of the cervical spine in the acute evaluation of blunt trauma. J Trauma. 2002;53:426–429 Pollack CV Jr, Hendey GW, Martin DR, Hoffman JR, Mower WR; NEXUS Group. Use of flexion–extension radiographs of the cervical spine in blunt trauma. Ann Emerg Med. 2001;38:8–11	Page: 83, 83 References: NA
